# Identification of Signaling Protein Complexes by Parallel Affinity Precipitation Coupled with Mass Spectrometry

**DOI:** 10.4137/CCI.S12484

**Published:** 2013-09-11

**Authors:** Heng Lu, Qishan Lin, Jihe Zhao

**Affiliations:** 1 Burnett School of Biomedical Science, College of Medicine, University of Central Florida, Orlando, FL, USA; 2 University at Albany Proteomics Facility, Center for Functional Genomics, Rensselaer, NY, USA

**Keywords:** parallel affinity precipitation (PAP), mass spectrometry, tandem affinity purification (TAP), protein–protein interaction, hemagglutinin tag, Myc tag, co-immunoprecipitation (co-IP)

## Abstract

Protein–protein interactions play a pivotal role in both inter- and intra-cellular signaling. Identification of signaling protein complexes can thus shed important new insights into cell communications. We developed a parallel affinity precipitation protocol to overcome the disadvantages of the tandem affinity purification procedure, such as the potential disruption of target protein conformation, subcellular localization or function by epitope tags, the potential need of large amounts of cell culture or generation of stable cell lines, and relatively long duration the two-step precipitation takes. This new simplified assay of protein interaction is quick, economic and specific. This paper describes the details in the design and method of the assay.

## Introduction

**Figure 1:**
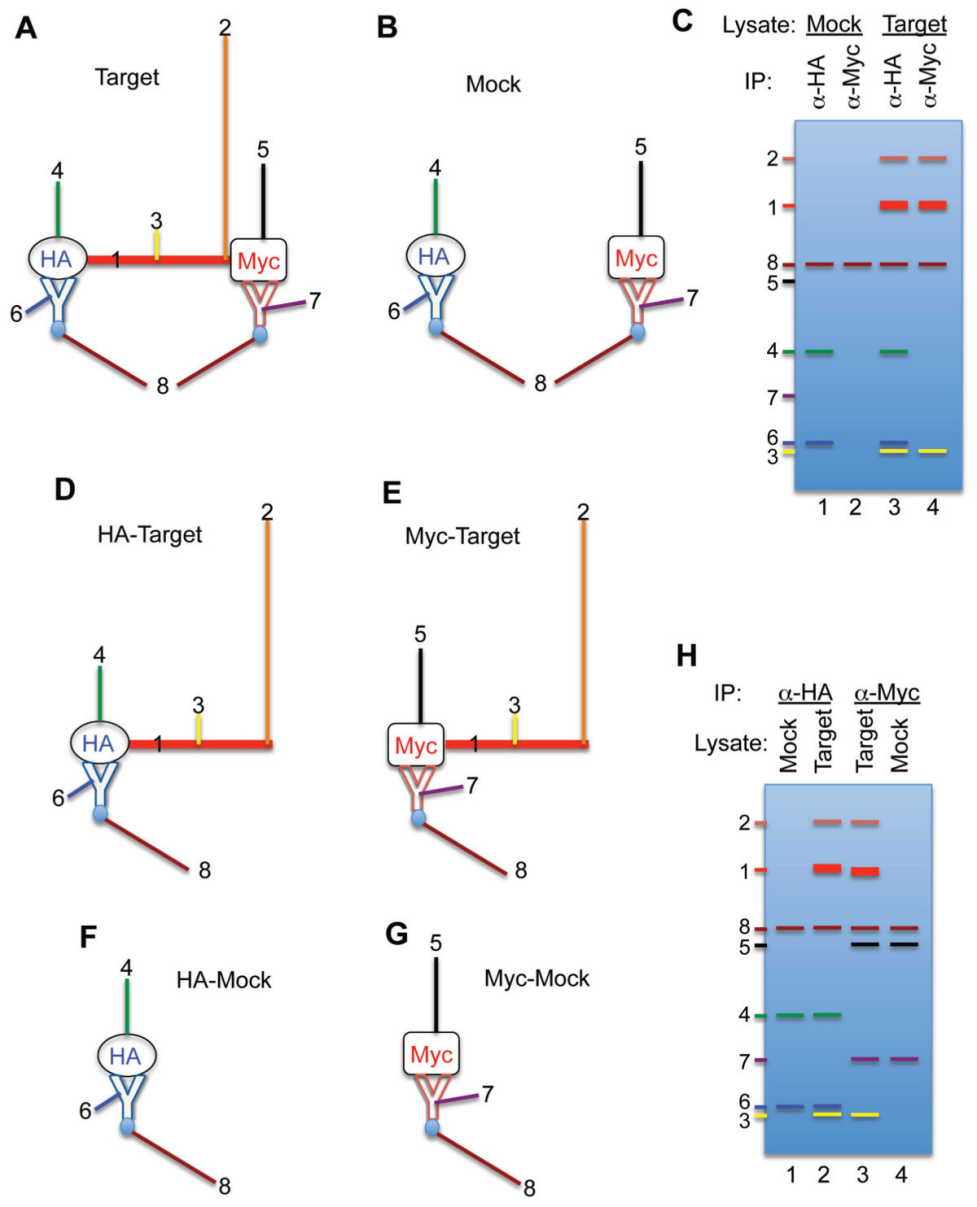
Schematic illustration of TAP (A–C) and PAP (D–H) assays In TAP, assume that the double tagged (HA and Myc in this case) target protein (#1, red) interacts specifically with two proteins (#2, brown; #3, yellow) in the target protein over-expressed cells (Target) (**A**). Control cells (mock) express the HA and Myc tags only (**B**). Also assume that non-specific interactions occur between protein #4 (green) and the HA tag, protein #5 (black) and the Myc tag, protein #6 (blue) and the anti-HA antibody (Y-shaped in blue), protein #7 (purple) and the anti-Myc antibody (Y-shaped in red), as well as protein #8 (brown) and the beads (solid circle in light blue). If the sequential precipitations are done first using the anti-HA antibody-conjugated beads followed by the anti-Myc antibody-conjugated beads, all the interacting proteins, specific or non-specific, would be precipitated by the first anti-HA IP except for proteins #5 and #7 which would be washed off prior to the second anti-Myc IP (**C**; lanes 1 and 3). The anti-Myc IP would then pull down the protein #8 only (**C**, lane 2) or the protein #8 together with the target protein and its specific interacting proteins #2 and #3 while leaving behind the non-specific proteins #4 and #6 (**C**, lane 4). In contrast, in PAP the two tags are added to the same end of the target protein (N-terminus in this case) to create two separate, single-tagged target protein constructs. Cells transiently over-expressing each of the target proteins (**D**, HA-Target and **E**, Myc-Target) or the tags only (**F**, HA-Mock and **G**, Myc-Mock) are used for a single step IP. The specific interacting proteins (#2 and #3) along with the target proteins would be pulled down by both antibodies only from the target cells (**H**, lanes 2 and 3) but not from the mock cells (**H**, lanes 1 and 4). **Notes:** The close distance between the Myc tag and the protein #2 binding site on the target protein (A) could jeopardize the interaction leading to failure to identify it. There is likely a need in TAP for transfecting two control vectors, one encoding the HA tag and the other encoding the Myc tag to make the mock cells (**B**). Protein #8 could be removed by pre-clearing IP using the antibody-free beads. In fact, the pHAN vector provides a Myc-His tag fusion. In this case, a single pHAN-target construct should suffice if specific precipitation using nickle-beads works as well. A protein band of a single molecular size does not necessarily mean a single protein identity in the band; more often than not, a single band contains more than one protein identity of the same molecular size. A target-protein binding protein could also weakly interact non-specifically with the tags, antibodies or beads, in this case the protein would be precipitated in a larger amount (thicker band) from the target cells than from the mock cells. Proteins interacting indirectly with the target protein are not drawn for fear to complicate the illustration. **Abbreviations:** IP, immunoprecipitation; PAP, parallel affinity precipitation; TAP, tandem affinity purification.

We developed the parallel affinity precipitation (PAP) coupled with mass spectrometry assay for quick identification of proteins that interact specifically with Krüppel-like factor 8 (KLF8) in mammalian cells^1^ by modifying the tandem affinity purification (TAP) method. TAP was originally developed for purification of protein complexes from yeast.^2–4^ TAP soon found increasingly wide application for isolation of protein complexes from mammalian cells.^5–7^ TAP is based on two-step sequential co-precipitations of protein complexes using antibodies or affinity beads specific for one of two different epitope tags one of which is linked to the amino terminus and the other to the carboxyl terminus of the target protein (or bait protein). After resolved on a protein gradient gel, the identities of the target protein co-precipitated proteins (gel bands) are determined by mass spectrometry. The two-step precipitations plus a protein cleavage and/or protein complex elution step was designed in TAP to minimize the inevitable non-specific pull-down of irrelevant proteins. This design, however, often requires large amounts of cell lysate (from up to several liters of cell culture) and generation of a cell line stably expressing the target protein to start with. Another disadvantage of TAP is that tagging both termini of a target protein sometimes interferes with the target protein conformation, function, and even cellular localization resulting in identification of false complexes associated with the target protein. This is particularly true in the case of proteins like KLF family proteins if used as target proteins. Given their conserved DNA binding domain at the very carboxyl terminus, a tag to the carboxyl terminus of a KLF family protein will likely alter their protein interaction profile, if not nuclear localization, and even function. For these reasons, we developed the PAP assay. In the PAP assay, the target protein is tagged on only one of the two termini, which is less likely to interfere with function, conformation, or subcellular localization of the target protein. Both a HA-tagged target protein and a Myc-tagged target protein are generated. After transiently overexpressed, these tagged proteins and their associated protein complexes are co-immunoprecipitated (co-IPed) in parallel using anti-HA and anti-Myc antibody-conjugated beads, respectively. After resolving on a protein gradient gel, only the bands that are shown in both the anti-HA and anti-Myc precipitates but absent in the precipitates from both of the mock controls are collected for mass spectrometry. We found that PAP assay requires significantly less cells/lysates and saves large amounts of time without compromising specificity of the complex isolation. Since whole cell lysates are used, this method is suitable for identifying the interaction between any proteins, be they membrane, cytosolic, or nuclear. The TAP and PAP assays are schematically illustrated side-to-side in Figure 1.

## Flow Chart

Construct hemagglutinin (HA)-tagged and Myc-tagged target protein plasmids


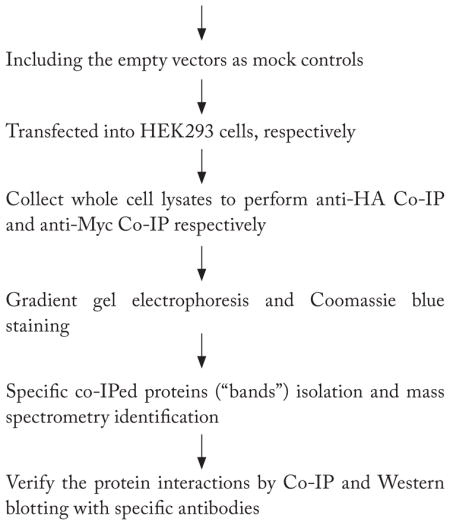


## Materials

### Polymerase chain reaction (PCR) and deoxyribonucleic acid (DNA) agarose gel electrophoresis

1. pKH3 mammalian expression vector (for HA-tag);^1,7–10^
2. pHAN mammalian expression vector (for Myc-tag);^1,11,12^
3. Deep Vent DNA polymerase (#M0258S; New England Biolabs, Ipswich, MA, USA);
4. T4 DNA ligase (#M0202S; New England Biolabs);
5. Aurum Plasmid Mini Kit (#732-6400; Bio-Rad Laboratories Inc, Hercules, CA, USA);
6. QIAquick Gel Extraction Kit (#28704; Qiagen, Germantown, MD, USA);
7. QIAprep Spin Mini-Prep Kit (Qiagen, #27104);
8. Eppendorf Thermal Cycler (Eppendorf North America, Hauppauge, NY, USA);
9. ChemiDoc^™^ XRS+ Image System (Bio-Rad);
10. 1 kb Plus DNA Ladder (Invitrogen, #10787-018);
11. Ampicillin (Fisher Scientific, #BP1760-5).

### Cell culture and transfection

1. HEK293T cells (#CRL-3216^™^; ATCC®, Manassas, VA, USA); Note, other cell types can be used depending upon the experimental need, preferably transfectable human or mouse cell lines because the mass spectrometry data base have all the human and mouse sequences and may not be fully available for other species;
2. Lipofectamine 2000 Transfection Reagent (#11668-019; Invitrogen, Carlsbad, CA, USA); In case this reagent is toxic to the cell line to be used, less toxic transfection reagents such as X-treme GENE from Roche and Fugene 6 from Promega can be used;
3. Dulbecco’s modified eagle medium (DMEM; #11965-092; Invitrogen);
4. Falcon 100 mm tissue culture dishes (#353003; Fisher Scientific, Waltham, MA, USA).

### Co-immunoprecipitaion

1. Nonidet P-40 (NP-40) lysis buffer with protease inhibitors purchased from Fisher Scientific: 20 mM Tris (#BP152-5), pH 8.0, 137 mM NaCl (#BP3581), 1% NP-40(#P128324), 10% glycerol (#G33-1), 1 mM Na3VO4 (#S454-50), 1 mM phenylmethylsulfonyl fluoride (#ICN800263), 10 mg/ml aprotinin (#BP2503-10) and 20 mg/ml leupeptin (#BP2662-5);
2. Anti-HA (A2095) and anti-Myc (A7470) antibody-conjugated agarose beads (Sigma-Aldrich, St Louis, MI, USA).

### Gradient Tris-HEPES gel electrophoresis and Western blotting

1. Four to twenty percent Tris-HEPES gradient mini gel (#NH21-420; NuSep, Bogart, GA, USA);
2. TruSep sodium dodecyl sulfate (SDS) Sample Buffer 5 mL × 2 (2×; #BG-145; NuSep);
3. Tris-HEPES/SDS running buffer (#BG-163; NuSep);
4. Precision Plus Standard Dual Color protein molecular weight markers (#161-0374; Bio-Rad; Note, this markers cover a range between 10 and 250 kD;
5. Horseradish peroxidase (HRP)-donkey anti-mouse IgG (H + L; #017-000-003; Jackson ImmunoResearchLaboratories, Inc, West Grove, PA, USA) with a 1:5,000 dilution for secondary antibody incubation in Western blotting;
6. PierceEnhanced Chemiluminescent (ECL) Substrate (#32106; Thermo scientific, Rockford, IL, USA);
7. ChemiDoc^™^ XRS+ Image System (Bio-Rad).

### Coomassie blue stain

1. Coomassie blue stain reagent (Bio-Safe; #161-0786; Bio-Rad);
2. Acetic acid (#A35-500; Fisher Scientific);
3. Ethanol (#A406F-1GAL) and methanol (#A41220) from Fisher Scientific;
4. Digital scanner (HP, Palo Alto, CA, USA).

### Equipment for mass spectrometry

1. Fused silica capillary (Polymicro Technologies, Tucson, AZ, USA);
2. Vydac Everest C18 packing material, 5 μm with 300 A pore (#238EV C18; Grace Vydac, Hesperia, CA, USA);
3. Microcapillary puller (model P-97, Sutter Instrument, Novato, CA, USA);
4. AB SCIEX QSTAR XL electrospray tandem mass spectrometer (AB SCIEX, Toronto, ON, Canada);
5. High-performance liquid chromatography (HPLC), CapLC (Waters, Milford, MA, USA);
6. Autosampler, FAMOS, (LC Packings, San Francisco, CA, USA).

### Reagents for mass spectrometry

1. HPLC buffer A: 0.1% formic acid (#27001, Sigma-Aldrich, St. louis, MO, USA), in Milli-Q water and 3% acetonitrile (#AX0145-1, EMD Millipore, Bellerica, MA, USA); HPLC buffer B: 0.1% formic acid in 85% acetonitrile, 10% isopropanol (#650447-4L; Sigma-Aldrich) and 5% Milli-Q water.
2. HPLC methanol grade (#9093-33; Avantor Performance Materials, Inc, Center Valley, PA, USA).

## Methods

1. Construct two target protein expression vectors, one with an HA tag and the other with a Myc tag:
  1.1 Design primers with appropriate restriction enzyme digestion sites, and amplify target protein cDNA from cDNA cloning vector;
  1.2 Use appropriate restriction enzymes to digest the cDNA fragment, then purify and insert them into the expression vectors, pKH3 and pHAN, respectively. These two vectors share identical cloning sites making the plasmid construction convenient, particularly for generating N-terminal HA or Myc tagged proteins. However, if a C-terminal HA or Myc tagged protein is preferred, there are also some cloning sites upstream of the tag peptide that can be used;
  1.3 Transfect each of the two vectors at 2 μg/well into HEK293T cells grown in falcon tissue culture 6-well plates. After an overnight incubation, collect the whole cell lysates (WCLs) for Western blotting to verify the expression of the HA- or Myc-tagged target proteins.
2. Co-immunoprecipitation:
  2.1 Overexpress the HA-protein or Myc-protein by transfecting the pKH3-target or pHAN-target vectors at 12 μg/well into HEK293T cells in falcon 100-mm tissue culture dishes; pKH3 or pHAN empty vectors are transfected as mock controls;
  2.2 After a 48-hour incubation, collect WCLs in 1 mL/dish of the NP-40 lysis buffer supplemented with protease inhibitors on ice;
  2.3 Add 50 μL of the NP-40 lysis buffer pre-washed anti-HA or anti-Myc antibody-conjugated agarose beads to 1 mL of the WCLs. Add the same amount of the beads to the mock control WCLs;
  2.4 Incubate overnight at 4°C on a rotation rack. Centrifuge at 8000 g to collect the precipitates;
  2.5 Wash the precipitates with the NP-40 lysis buffer 3 times at 4°C, then add the TruSep SDS Sample buffer (50–200 μL) and denature precipitated proteins at 95°C for 10 minutes.
3. Resolve the precipitated protein samples by gradient Tris-HEPES gel electrophoresis:
  3.1 Parallel-load (in the order of molecular weight marker, HA mock, HA-Target, Myc-Target, Myc mock, molecular weight marker; see Fig. 1H) the co-IPed protein samples (10–50 μL) into 4%–20% Tris-HEPES gradient mini gels previously described,^1^ load dual color protein markers on both sides of the gel for molecular weight indicator;
  3.2 Run the gel for 60 minutes at 100 volts or until the dye front gets close to the bottom of the gel.
4. Coomassie blue staining:
  4.1 Wash the gel for 5 minutes three times with distilled, deionized nano-Q water (ddH_2_O) at room temperature;
  4.2 Fix the gel with the gel fixation buffer containing 40% methanol, 10% acetic acid, and 50% ddH_2_O for 60 minutes;
  4.3 Rinse the gel briefly with ddH_2_O three times;
  4.4 Wash the gel with 50% ethanol in ddH_2_O at 4°C for overnight to decrease the gel background;
  4.5 Rinse the gel briefly with ddH_2_O three times at room temperature;
  4.6 Stain the gel in Bio-Safe^™^ Coomassie stain solution on a rocker by gently rocking for 1 hour;
  4.7 Rinse the gel briefly with ddH_2_O three times at room temperature;
  4.8 Wash the gel with ddH_2_O overnight at room temperature;
  4.9 Wrap the gel with a piece of clean Saran wrap. Scan the gel for the image of the protein bands (note, never touch the gels with bare skin to avoid keratin contamination);
  4.10 Keep the gel in 1%–2% acetic acid at room temperature.
5. Select potential interacting proteins (bands) of the target protein:
  5.1 Compare the HA-protein and Myc-protein lanes to select the protein bands of the same molecular weights that appear in both of the target lanes but are not visible or appear as much lighter bands in either of the mock lanes. Collect also the target protein bands for identity verification (Fig. 1).
6. In-gel tryptic digestion:
  6.1 Excise the bands of interest in both HA-protein and Myc-protein lanes for mass spectrometry analysis;
  6.2 Cut the gel into small pieces and wash the pieces in 50% Acetonitrile, 100 mM NH_4_HCO_3_ to remove residual SDS or Coomassie blue;
  6.3 Shrink the gel pieces in acetonitrile and dry the gel pieces in Speed-Vac;
  6.4 Swell the gel pieces in a digestion buffer containing 50 mM NH_4_HCO_3_ and 12.5 ng/μL of trypsin (Sigma, Proteomics grade) in an ice-cold bath for 45 minutes;
  6.5 Remove the extra supernatant;
  6.6 Replace with 5–10 μL of the digestion buffer (50 mM NH_4_HCO_3_, 5 mM CaCl_2_);
  6.7 Keep the gel pieces wet during enzymatic cleavage (37°C, overnight);
  6.8 Extract the cleavage products (peptides) by one change of 20 mM NH_4_HCO_3_;
  6.9 Spin, remove, and transfer the supernatant to a new Eppendorf tube;
  6.10 Further extract the cleavage products for 3 more times using 5% formic acid plus 50% acetonitrile at the room temperature;
  6.11 Pool the supernatants together;
  6.12 Speed-Vac dry the supernatant until the volume is 1–2 μL.
7. Protein identification by electrospray tandem mass spectrometry:
  7.1 Both the trap column (500 μm inner diameter (ID) × 2.54 cm outside diameter (OD) × 15 mm) and analytic column (100 μm ID × 365 μm OD × 150 mm) are packed with an Everest C18 resin (5 μm, Grace Vydac);
  7.2 One end of the analytic column is pulled into a PicoFrit using a Sutter Instrument laser puller and placed directly into the QSTAR XL NanoSpray II source, limiting the peak dispersion after the column. The other end of the column is connected to the 10-port valve stream selector through a conductive Micro Tight union (Sigma-Aldrich) using 25 μm ID × 365 μm OD × 8 cm capillary tubing;
  7.3 A Waters CapLC system consisting of an autosampler, A, B, and C pumps and a stream selector is used for desalting and gradient formation. The pump outlet is connected to the 10-port valve through a splitting tee that reduces the flow from the pump from 9 μL/minute to 300 nL/minute using a length of restriction tubing made from fused silica;
  7.4 Contact closure connection is made between the autosampler and the mass spectrometer;
  7.5 Waters/Micromass Masslynx 3.5 software is used to program the CapLC system. The peptide mixture (50 μL) is injected and desalted on the trap column for 6 minutes and eluted onto the analytic column at 300 nL/minute by the application of a series of mobile phase B gradients (5%–10% B for 4 minutes, 10%–30% B for 61 minutes, 30%–85% B for 5 minutes, 85% B for 5 minutes, and 10% B for 6 minutes);
  7.6 The mass spectrometer QSTAR XL is controlled through Analyst 1.1 (ABSCIEX, Framingham, MA, USA) software and operated in an information dependent acquisition mode whereby, following the interrogation of mass spectrometry (MS) data, ions are selected for MS/MS analysis based on their intensity (10 cps) and charge state +2, +3, and +4. Rolling collision energies are chosen automatically based on the m/z and charge-state of the selected precursor ions. An mass spectrometry-time of flight (MS-TOF) survey scan is from m/z 350–1,300 with an acquisition time of 1 second. The three most abundant ions present in the survey spectrum are automatically mass-selected and fragmented by collision-induced dissociation (3 seconds per MS/MS spectrum);
  7.7 The MSMS data (wiff file) are converted to a Mascot generic file format using a Mascot “script” 1.6b27 for the database search;
  7.8 MASCOT 2.3 from Matrix Science (London, UK) is used to search all the tandem mass spectra against NCBI non-redundant human sub-database. The parameters used for the searches are as follows: fixed modification including carbamidomethylation for cysteine residueand variable modifications including methionine in oxidized form and deamidation (NQ) are considered. If negative hits are obtained from the search, de novo sequencing (manually spectrum interpretation) will be performed. With the mass spectrum analyzer’ help, you can log into MASCOT 2.2 for Peptide Mass Fingerprint, Sequence Query, or MS/MS Ion Search services. Also, you can get more information and help from the MASCOT website (http://www.matrixscience.com/search_form_select.html); Compare protein identities of the two bands at the same molecular weight from HA-protein and Myc-protein lanes; an overlap between two identity groups indicates highly potential interacting protein candidates.
8. Troubleshooting (see Table 1).

## Figures and Tables

**Table 1:** Troubleshooting guide.

STEP	PROBLEM	SOLUTION
1	Western blotting indicates low expression levels of the tagged target proteins	Increase the amounts of reagent, such as the plasmids
4	Coomassie blue stain shows weak staining of the tagged target proteins	This is likely due to the loss of proteins during the Co-IP process. Optimize the Co-IP conditions by using less stringent lysis buffer, more antibody-conjugated beads, or centrifuge at higher speed and/or for longer time during washing
4	Strong tagged target protein bands, but few Co-IPed protein bands	Restart with more cells to collect larger volume of cell lysates; Improve the Co-IP conditions, pay special attention to buffer pH
4	Strong tagged target protein staining and clear Co-IPed protein bands, but high background in controls	Follow rigorous Co-IP washing process; pre-clean lysates by adding greater amount of control agarose beads; reduce the concentration of detergent or NP-40
6	High noises of mass spectrum	Follow rigorous gel operation process; excise bands with smallest margin possible
6	Over-abundant keratin signals	Handle gels/bands with extreme caution to avoid direct skin contact with gels/bands

**Abbreviation:** Co-IP, co-immunoprecipitate.
